# Indonesian healthcare professionals’ experiences in rural and urban settings during the first wave of COVID-19: A qualitative study

**DOI:** 10.1371/journal.pone.0288256

**Published:** 2023-07-11

**Authors:** Ida Ayu Sutrisni, Aria Kekalih, Dewi Friska, Diana Timoria, Ralalicia Limato, Ragil Dien, Claus Bogh, Mary Chambers, Sonia Lewycka, Jennifer Ilo Van Nuil, Raph L Hamers, on behalf of the OUCRU COVID-19 Research Group

**Affiliations:** 1 Faculty of Medicine Universitas Indonesia, Oxford University Clinical Research Unit Indonesia, Jakarta, Indonesia; 2 Faculty of Medicine Universitas Indonesia, Department of Community Medicine, Jakarta, Indonesia; 3 Sumba Foundation, Sumba, Indonesia; 4 Nuffield Department of Medicine, Centre for Tropical Medicine and Global Health, University of Oxford, Oxford, United Kingdom; 5 Oxford University Clinical Research Unit, Ho Chi Minh City, Vietnam; 6 Oxford University Clinical Research Unit, Hanoi, Vietnam; School of Health Binawan: Universitas Binawan, INDONESIA

## Abstract

**Introduction:**

During the COVID-19 pandemic, healthcare workers (HCWs) faced unprecedented challenges, increased workload, and often struggled to provide healthcare services. We explored the experiences faced by HCWs working at primary healthcare centers (PHCs) and hospitals across urban and rural settings in Indonesia.

**Methods:**

As part of a larger multi-country study, we conducted semi-structured in-depth interviews with a purposive sample of Indonesian HCWs. We used thematic analysis to identify the main challenges described by the participants.

**Results:**

We interviewed 40 HCWs between December 2020 and March 2021. We identified that challenges varied depending on their role. i) For those in clinical roles, challenges included maintaining trust with communities, and patient referral issues; ii) for those in non-clinical roles, sub-optimal laboratory capacity and logistics, and lack of training were the main challenges; iii) for managerial roles, challenges included access to budget and supplies, and staff shortages due to isolation and overwork. There were also several cross-cutting challenges across all the roles including limited or rapidly changing information (in urban settings), and culture and communication (in rural settings). All of these challenges contributed to mental health issues among all HCW cadres.

**Conclusions:**

HCWs across roles and settings were confronted with unprecedented challenges. Understanding the various challenges across different healthcare cadres and within different settings is crucial for supporting HCWs during pandemic times. In rural areas, in particular, HCWs should be more sensitive to cultural and linguistic differences to enhance the effectiveness and awareness of public health messages.

## Introduction

Low and middle-income countries (LMICs) have been disproportionately impacted by severe acute respiratory syndrome coronavirus 2 (SARS-CoV-2), the virus causing COVID-19. During the early stages of the pandemic, diverse challenges faced by healthcare workers (HCWs) included a lack of accurate information regarding disease management [1], limited availability of adequate personal protective equipment (PPE) [2], limited access to diagnostic tests [2], and psychosocial factors such as unsupportive work environments, excessive workload, and a lack of time to rest for frontline HCWs [2–4].

As of October 10th, 2022, there have been an estimated total of 6.4 million confirmed cases and 158 thousand deaths [5], ranking Indonesia as the second worst affected country in the Asian region. Since March 2020, Indonesia experienced an extended first wave that steadily trended upward through January 2021, a Delta-driven second wave in July 2021, and an Omicron-driven third wave in January 2022. The highest number of reported cases occurred in the major cities on Java Island, although COVID-19 also spread widely among remote, rural communities across the vast Indonesian archipelago. During the pandemic, the health system struggled to manage the number of patients as the virus kept spreading and frontline HCWs with direct contact with COVID-19 patients were at a higher risk of infection [6]. The health system appeared ill-prepared to deal with the pandemic, making HCWs a vulnerable target for nosocomial infection, with a five-fold risk of dying from COVID-19 compared to the general population [7]. Since the start of the pandemic, approximately 2,087 HCWs died due to COVID-19, with the highest reported deaths among doctors, midwives, and nurses between March 2020 and October 2022 [8]. A small study early in the pandemic among medical hospital staff in the city of Surabaya, reported feelings of physical and psychological exhaustion during COVID-19 for a variety of reasons (e.g. limited staff, length of diagnostic procedures, limited skills, and shortage of beds) [9]. However, little is known about the specific challenges faced by HCWS employed in different professional roles (e.g. clinical, non-clinical, managerial roles), settings (e.g. rural, urban), and healthcare system levels (e.g. hospital, primary care levels). As part of a multi-country social science and public engagement action research (SPEAR) project [10], we conducted a qualitative study, based on in-depth interviews (IDIs) with a range of HCWs, to explore challenges faced by HCWs working in clinical, non-clinical and managerial roles, and across different healthcare levels and geographic settings in Indonesia.

## Materials and methods

### Study design

SPEAR is mixed-methods social science and community engagement study using quantitative surveys, key informant discussions (KIDs), IDIs, and participant-led digital diaries across 13 sites in Indonesia, Nepal, and Vietnam. SPEAR is divided into two main phases, which is fully described in a protocol paper [10]. The overarching aim of SPEAR Phase 1 was to explore the lived experiences and impact of COVID-19 on HCWs, health-related staff, and vulnerable communities in 13 sites, while the aim of Phase 2 was to explore themes surrounding acceptance and accessibility of COVID-19 vaccines within the same settings. SPEAR data collection for Phases 1 and 2 took place from December 2020 to Jun 2022. In this paper, we describe data that were drawn from the Phase 1 qualitative IDIs and KIDs conducted with HCWs and related staff within the three sites in Indonesia.

### Study setting

Indonesia is a lower-middle income country in Southeast Asia with the world’s fourth largest population (estimated to be 275 million), with a wide range of socio-economic conditions and health indicators across the archipelago [11]. Indonesia has a decentralised public healthcare system in which provincial or district-level governments have authority over most public hospitals and primary healthcare centres (PHCs), and there is also a substantial private healthcare sector [12]. To achieve the goal of universal healthcare coverage, in 2014 the government introduced national health insurance (*Jaminan Kesehatan Nasional*), which had reached 84% of the population by 2021 [13]. The SPEAR study sites in Indonesia included both urban and rural settings: i) Jakarta city (urban), the national capital, a megacity of 10.6 million inhabitants with a total of 122 hospitals and 252 PHCs [14]; ii) Bandung city (urban), the capital city of West Java province, with a population of 2.4 million and total of 26 hospitals and 70 PHCs [15]; iii) and West Sumba (145,097 population, 2 hospitals and 10 PHCs) [16] and Southwest Sumba (303,650 population, 2 hospitals and 16 PHCs) districts (both rural), on the remote island of Sumba in East Nusa Tenggara province [17].

### Participant sampling and recruitment

We recruited key informants from study sites in order to gain perspectives on the situation at each site, as well as to identify potential participants for the subsequent data collection. Key informants were interviewed prior the IDIs to obtain contextual information and to gain advice regarding data collection and potential interviewees at each site. For the IDIs that are presented in this analysis, we used purposive sampling to recruit 40 participants who were working in hospitals and PHCs during the data collection period of SPEAR 1. We used participant mapping based on information from the survey including demographic characteristics (e.g., age, gender), job related specifics (e.g., position or role), and responses to specific questions of interest. Most participants (75%) were recruited from the mapping [10] while the remaining participants (25%) were recruited from leaders within the study sites based on the key informants’ advice. Participants were selected from five hospitals (three hospitals in Sumba and one hospital in Jakarta and Bandung each) and 15 PHCs (four in Jakarta, five in Bandung, and six in Sumba).

### Data collection

The data collection for Phase 1 qualitative component with HCWs and related staff in Indonesia took place between December 2020 and March 2021, which corresponded with the first wave of COVID-19 in Indonesia. Three Indonesian researchers conducted the interviews in the local language, either face-to-face, and following the COVID-19 guidelines, or virtually via video or voice calls. For the study tool for the IDI, we used an interview guide which covered topics related to perspectives and experiences working during the COVID-19 pandemic, the impact of COVID-19 to careers and work responsibilities, impacts of HCWs’ work on their relationships with family and community, and stigma and community responses around COVID-19 (S3 & S4). Prior to the interview, prospective participants were contacted and asked for their willingness and availability to be interviewed. Upon agreement, the interviewer arranged the time and venue/media of the interview. The interviewer provided information regarding the study, confidentiality, and how the data would be handled, disseminated, and used in the future before the interview. We then obtained written or oral informed consent from all the participants for the interview to take place and for it to be audio recorded. The interviewers supplemented the audio with handwritten field notes. We expected the interview duration to be between 60 and 90 minutes.

### Data analysis

The interviews were transcribed in the language spoken and translated into English. We removed identifying information during the transcription process to preserve the confidentiality of the participants [18]. We managed the transcripts in NVivo 12 software. For this analysis, we used an iterative process that included both deductive and inductive coding techniques. The SPEAR Indonesia team conducted multiple cycles of coding (IAS, DT, RD), with input and discussion from study investigators (JIVN, DF, AK, HRL, LS, CM). To start, we created a coding framework with deductive codes (i.e. topics that inform the study objectives, driven by the researchers) and inductive topics from the regular debrief sessions conducted with the full international SPEAR team during the data collection period [19–21]. During provisional coding, we also integrated additional categories and topics into the codebook and discussed during the debrief meetings with other sites. For the second cycle coding for this analysis, we used the ‘relevant text’ as coded from the first cycle of coding [22]. We then conducted pattern coding to explore the larger patterns and themes in the data [21]. We discussed the patterns and themes using an iterative process of discussion and refinement of the core themes over multiple meetings and compared between rural and urban sites, as well as between roles within the health centers. We then contrasted data between urban and rural sites as well as between hospitals and PHCs.

### Ethical approvals

The SPEAR study in Indonesia was reviewed and approved by Ethics Committee of the Faculty of Medicine, University of Indonesia (1283/UN2.F1/ETIK/PPM.00.02/2020) and Oxford Tropical Research Ethics Committee (547–20).

## Results

### Participant characteristics

A total of 40 participants between the ages of 20 and 59 years participated in the study including 26 females and 14 males, 25 from urban settings and 15 from rural settings, 7 working in hospitals and 33 working in the PHCs. 18 participants were working in clinical roles, 17 in non-clinical roles, and 5 in managerial roles (Table 1).

**Table 1:** Participant demographics.

Remarks	n (%) n = 40
Sex	
Female	26 (65)
Male	14 (35)
Settings	
Urban	25 (62.5)
Rural	15 (37.5)
Workplace	
PHC (Puskesmas)	33 (82.5)
Hospital	7 (17.5)
Age group	
20–29	7 (17.5)
30–39	16 (40)
40–49	5 (12.5)
≥50	3 (7.5)
Not stated	9 (22.5)
Participant professions roles	
Managerial roles	**5 (12.5)**
Hospital director	1 (2.5)
Head of PHC	2 (5)
Management staff	2 (5)
Medical roles	**18 (45)**
Doctors	5 (12.5)
Psychiatrist	1 (2.5)
Nurse	9 (22.5)
Midwife	3 (7.5)
Non-medical roles	**17 (42.5)**
Health promotion officer	4 (10)
Administrative staff	3 (7.5)
Laboratory staff	3 (7.5)
Nutritionist	1 (2.5)
Pharmacist	2 (5)
Spiritual officer	1 (2.5)
Tracing officer	1 (2.5)
Cleaners	2 (5)

We grouped the findings into four main categories based on primary job role including challenges in clinical roles, non-clinical roles, managerial roles, and cross-cutting challenges across all roles. We also noted if specific challenges related to both rural and urban settings or if the challenges were unique to either setting (Table 2).

**Table 2:** Summary of key elements/challenges faced by HCWs by roles.

Main themes	Key narrative elements/challenges
**Challenges in clinical role**	
Obtaining public trust	Challenges in performing duty of care, contact tracing as an example, is influenced by lack of trust among community (dishonest with travel history, quarantine refusal and distrust with COVID-19 test result)
Patient referral system	Overburdening of COVID-19 referral hospitals in urban sites led to stress and conflict between staff at PHCs, hospital level, and patient’s families.
**Challenges in non-clinical role**	
Limited trainings related to COVID-19 measures	Lack of communication training and limited time to transfer knowledge to other colleagues confronted during the pandemic Lack of training and mixed messages led to difficulty working collaboratively to perform contact tracing with local cross-sectors.
Sub-optimal laboratory and logistic capacity	Limited lab capacity and surge COVID-19 cases created a delay in PCR results for several days. In addition to rural sites, the sample needs to be sent to other islands, where have PCR laboratory.
**Challenges in managerial roles**	
Budget and supplies	Budget management, especially for PPE and medical waste disposal, was complex and often led to budget deficits.
Staff shortage due to isolation and overwork	Engaging and motivating health staff, with high numbers of staff having to isolate and die due to COVID-19
**Cross-cutting challenges across roles**	
Increasing mental health issues	Contributing factors on HCWs mental health include having infection, work overload, death among HCWs, shortage staff and fear to transmit COVID-19 to others, payment delays, and complex situations in coordinating contact tracing
Rapidly changing or lack of information	Health staff had difficulties providing updates and accurate information to the community because the information changed rapidly Frequent COVID-19 policy revisions influenced practice Staff need extra attention for rapid information on hospital space allocation for COVID-19 case management
Culture and communication barriers	Communication and explanation in local languages was more effective and understandable Dilemmas between cultural practices (such as nose kissing in Sumba) and COVID-19 prevention methods

### Reported challenges

#### 1. Clinical roles

For clinical roles, the main challenges reported included maintaining trust with communities and patient referral issues. The participants’ narratives often included feelings of frustration, fear, and stress.

*Maintaining community trust*. Building and maintaining community trust was a challenge in both urban and rural healthcare settings. HCWs reported a lack of openness from the community in some instances (e.g. dishonesty regarding travel history). This caused difficulties for HCWs to carry out their duties related to the pandemic, for example contact tracing. A nurse at the rural hospital described challenges related to people not sharing information about travel. From her point of view, the silence meant dishonesty.

*“A woman*, *she just arrived from Bali two days ago*. *She is originally from West Sumba*. *I asked them*, *do you have any family members who traveled outside the area these days*? *She didn’t say anything*.*"*

(13SUM1814, Female, Nurse, Rural Hospital).

In urban settings, lack of trust caused the quarantine process to be complicated and it often took time to urge individuals to go to an isolation facility. One HCW described a struggle when they picked up a pregnant woman, who contracted COVID-19, from her home to be transferred to the quarantine facility. Her family did not give consent for her release as she stated that she felt fine and did not believe the COVID-19 test result. In this case, the local leaders became involved with the family to help facilitate the process. This was a common occurrence across sites.

*“Many of them [patients] and their families refused to give birth in the referral hospitals*, *and they did not believe the COVID-19 test results*. *When the Puskesmas team went to her house to pick her up*, *she did not want to go out*.*”*

(26JKT1798, Female, Midwife, Urban PHC)

Similarly, HCWs based in rural sites mentioned that it was challenging when people did not believe the results of the COVID-19 tests or advice from the HCWs. It was not easy to inform people with positive COVID-19 results, particularly those who were asymptomatic.

*“A traveler was back from Jambi*, *he confirmed as a positive patient*, *but at first*, *his parents did not accept that their son was positive for COVID-19*.*”*

(20SUM953, Female, Midwife, Rural PHC)

*Patient referral issues*. Another challenge in the urban settings was related to referring COVID-19 patients to COVID-19 designated hospitals during first wave. The referral process was complicated because most referral hospitals were full at that time. HCWs at PHCs level were forced to contact many hospitals to refer each COVID-19 patient, which caused stress and conflicts between staff at PHCs, hospitals, and patients’ families.

*"We [PHC staff] cannot handle a pregnant mother with breech presentation at PHC*. *It was an extreme emergency situation*. *We called a hospital only telling them we are on the way to the hospital although they said the hospital capacity was full with many reasons*. *However*, *we kept going and had no choices*. *It was extremely challenging to find a hospital*, *even we have got a scolding from the hospital staff*, *so what I can say it was an emergency situation*.*”*

(26JKT1798, Female, Midwife, Urban PHC)

HCWs were facing a crisis situation in which it was extremely challenging to refer patients and find available hospital beds. The usual referral protocols were not followed, and HCWs had to improvise to help the patients.

#### 2. Non-clinical roles

For those working in non-clinical roles, the main challenges discussed in both settings were lack of training and sub-optimal laboratory and other logistic capacities.

*Lack of training related to the COVID-19 response*. Across all settings, participants described how there was minimal training given in response to the pandemic. This related to a combination of limited staff to provide the trainings and not enough time to conduct formal trainings.

*“… the challenging part was when doing the swab on the nose and the throat*. *We must learn a lot about it through training*. *Indeed*, *we had our seniors who had received some training but that was only 2 people*. *We are still novices*, *learning a little bit from them*, *and we haven’t received training*.*”*

(22SUM1727, Male, Laboratory staff, Rural Hospital)

Participants also mentioned that they received no training on good communication practices.

*“When we were assigned as a tracer*, *we try our best to do it ourselves*. *Like in terms of communication*, *how to deliver information to the patient’s family and the patient himself… I learned it by myself*. *Because there hasn’t been any training about it [communication]*.*”*

(29JKT1743, Male, Administrative staff, Urban PHC)

Importantly, communication breakdowns impacted the collaborative work between the COVID-19 task force and the HCWs. For example, the management of the isolation process for individuals with asymptomatic COVID-19 was exceptionally challenging. One participant reported that a task force member ignored the guidelines that those with asymptomatic COVID-19 should also be isolated.

*"The patient did not report to us [one of covid-19 taskforce members]*, *he is still walking around normally*. *Then if you report to me*, *what should I do*?*" [participant was repeating the local team response]*.*”*

(16BDG2870, Female, Tracer, 24)

*Limited laboratory and other logistics capacity*. Early into the pandemic, staff in non-clinical roles in both settings were overwhelmed with rising numbers of cases but were receiving little guidance on the COVID-19 swab procedures and were struggling with limited COVID-19 testing lab facilities. During the first wave, participants from the urban sites mentioned how the limited lab capacity for testing was linked to low number of COVID-19 cases detected.

*“From March to August*, *we only added 8 more COVID-19 patients*, *nothing more*. *Why*? *Because month per month starting March up to Mid-August there was one lab*… *an additional lab didn’t exist yet*. *We were still doing COVID-19 test with the provincial health office*. *Too complicated…”*

(10BDG2921, Male, Nurse, Urban PHC)

Similarly, in the rural health settings the laboratory facilities were not strengthened. Participants said they needed to wait more than one week to get the swab test results released because the samples were sent to another island with more laboratory facilities.

*“[COVID-19 test sample] need to be sent to Kupang [another island]*.*”*

(22SUM1727, Male, Laboratory staff, Urban PHC)

#### 3. Managerial roles

The pandemic challenges faced by those working at managerial levels included budget and supply challenges, medical waste management, and recruiting and retaining staff.

*Budget and supplies*. Challenges in securing and managing budgets (including audits) during the pandemic was a common concern in all the study sites. Managers struggled to purchase sufficient amounts of protective equipment (PPE) within their budgets.

*“If we order tens of thousands of PPE*, *the standards are not the same*, *some are good*, *many are not*. *What is not good*, *this cannot be used by health workers*.*”*

(39JKT, Male, Director, Urban Hospital)

*“We didn’t have a stock of KN95 masks*. *It’s a standard mask for health workers who are assigned in the isolation room*. *It happened for around 2 weeks*, *it’s not available anywhere*. *I asked the hospital to purchase it*, *but the hospital said don’t have budget*.*”*

(12SUM1258, Female, Pharmacist, Rural Hospital)

In the early stages of the pandemic, staff in the urban settings reported shortages of PPE, including face masks. Many participants discussed how they would use raincoats or make their own PPE. Some sites received donations of PPE.

*Medical waste management*. Apart from dealing with the huge budget on infectious waste, participants also commented that medical waste management quickly became a challenge in both rural and urban settings. The increasing number of disposable PPE meant increases in medical waste from health facilities. Participants from the rural setting mentioned that although medical waste management was handled through a third party, the waste transportation was affected during the pandemic. As a result, the storage of medical waste onsite at the clinic became an issue, and managers needed extra space to store it before transporting it to the third-party company.

*“The waste became a problem because it was not directly thrown away*. *At that time*, *we still kept it near the toilet rear*. *It means it’s not safe*, *it should be directly destroyed*.*”*

(07SUM1975, Female, Pharmacist, Rural PHC)

*“Our medical waste is greatly increased*. *Those ingredients increase by tons in a year*, *uhm… a month*. *To burn them take a lot of money*, *it’s really extraordinary*.*”*

(39JKT, Male, Director, Urban Hospital)

*Staff sortage due to isolation and overwork*. Staff in management levels, primarily in urban sites, relayed challenges related to staff shortages and keeping staff engaged and motivated. Additional health staff often needed to be secured during the pandemic. Although the staff were equipped with PPE, many of them still contracted COVID-19 and the remaining staff had to cover for them during their absence. Participants equated dealing with COVID-19 with fighting on the battlefield, with no days off. In their minds, exposure to COVID-19 was unavoidable.

*“We share the load*, *the PHCs do not stop in giving care*. *At the moment I have 15 staff members suffering from COVID-19*.*”*

(23BDG2902, Head of PHC, Urban PHC)

*“Although we are already equipped with adequate PPE… There are still some friends*, *colleagues who contracted the virus*.*”*

(17JKT1869, Male, Doctor, Urban PHC)

Eventually, some wards or health units were closed due to staff shortages. One participant expressed the opinion that it was likely that these staff were infected with COVID-19 from outside the health facilities.

*“We closed several rooms… Because at that time more than half of the midwives and nurses were tested positive*. *But after being traced*, *the transmission was not from the patients*. *They got the transmission from friends*, *neighbors*.*”*

(36BDG3241, Male, Research and Training Unit, Urban Hospital)

Indeed, those in management roles felt fear and stress but also struggled as to how to keep their staff motivated and supported.

*“The challenging thing is that COVID cases have been increasing*, *and we have to make right decisions*. *The concern is how to tackle COVID-19 properly*, *securing [protecting HCWs from transmission and keep them motivated facing high cases] the workers is hard…*. *And last*, *the thing we are most afraid of is that our employees will burn-out*. *So*, *when they got fed up with the job*, *I can’t do anything else*. *Sometimes ago*, *the Health Office issued a rule that at this time of COVID-19 surge*, *no employees take on leave*.*“*

(39JKT, Male, Director, Urban Hospital)

#### 4. Common challenges across roles

Many of the challenges that health care workers faced were the same regardless of their role or setting. These cross-cutting challenges included mental health challenges, limited or rapid changes in information related to COVID-19 protocols, and language and cultural dilemmas in regard to communicating COVID-19 information.

*Increasing mental health issues*. Participants expressed fear, confusion, exhaustion, guilt, feeling overwhelmed, struggles, and at times felt unmotivated in their job roles. Often these descriptions were in the context of increased workload and limited human resources. In the urban settings, where incidence and mortality rates were higher, participants expressed fear over death of their colleagues, feeling overwhelmed due to increased workload and shortage of HCWs therefore time off was not always guaranteed. Further, participants spoke of feeling demotivated due to delayed incentives or extra remuneration as a financial aid given by government.

*“…currently*, *there are a lot of health workers who have been confirmed to have COVID-19*, *and lost their life*.*”*

(15JKT1852, Female, Doctor, Urban PHC)

*“We cannot relax*, *there is no day off*, *no leave*, *no sick leave*. *We continue to carry out the roles*.*”*

(02BDG2777, Male, Nurse, Urban PHC)

In the rural settings, participants described concerns over disease transmission to their family members or being infected by co-workers who had just arrived back from travelling. The fear was heightened because COVID-19 tests were not routinely performed in the rural areas.

*“The fear among fellow health professionals is*, *for example*, *there is a colleague who just came home from a trip*, *we were worried whether the person was infected or not*. *After self-isolation*, *the swab procedure was not performed*.*”*

(07SUM1975, Female, Pharmacist, Rural PHC)

In addition, participants reported feeling restless and overwhelmed when faced with complex situations in coordinating contact tracing. For instance, the manager was often contacted in the middle of the night to solve COVID-19 related cases and had to respond quickly as part of contact tracing. Then, due to the extra work, those in non-clinical roles (e.g. nutritionist) were also involved in aspects of COVID-19 coordination on tracing.

*"At midnight [outside working hours]*, *I got a call asking me to check [on] someone [who] passed away [at home] whether they have COVID-19 or not*!*…"*

*(23BDG*, *Female*, *Head of Puskesmas*, *Urban PHC)*

*"My responsibilities are actually getting heavier… I have a lot of work*, *…coordination for getting COVID-19 patients’ data and isolation process*."

(06SUM1951, Male, Nutritionist, Rural Hospital)

*Rapidly changing or lack of information*. Participants both in rural and urban settings expressed confusion regarding the rapidly changing information, guidelines, and procedures during the pandemic. Clinical staff mentioned the rapid changes in clinical guidelines while laboratory staff stated how the protocols to collect and handle COVID-19 specimens changed.

*“This puzzled us continuously why we only had 8 patients in all those months*. *This link to COVID-19 SOP 1*^*st*^, *2*^*nd*^, *and 3*^*rd*^ *revision about limited swab test at PHC [performed]*. *Then*, *after 5*^*th*^ *COVID-19 protocol revision used*, *more the COVID-19 cases are found since more testing was applied at PHC*.*”*

*(10BDG2921*, *Male*, *Nurse*, *Urban PHC)*

Further, all these rapid changes created difficulties for the HCWs to communicate the most up to date and accurate COVID-19 information to the community.

*"Technical instructions also change very quickly*. *For example a registration link for the COVID -19 vaccine provided for the elderly changed within a day—so that makes it difficult for us to send information regarding the registration link*. *So people ended up doing vaccine registration on the spot*, *which make staff work hastily*.*"*

(11JKT, Female, Doctor, Urban PHC)

*"We are confused*, *a few days ago we were given a policy update… oh now it has changed again…we need confirmation because sometimes we don’t understand a thing*, *suddenly there is a new policy that we need time to understand again*."

(21BDG2773, Female, Health Promotion Staff, Urban PHC)

Beyond the rapid changes in information, there were also other rapid changes, such as the transformation of hospital spaces into designated COVID-19 spaces, which required additional management.

*“This is a new hospital*, *there is no system available yet*. *When I joined this hospital*, *it just had its inauguration*. *So*, *I was the one who established the system here by creating workflow and set up the job description for each staff in this unit*. *I also deal with new staff*, *some of them are fresh graduates and lack experience…That’s the challenge*.*”*

(12_SUM_1258, Female, Pharmacist, Rural Hospital)

Participants informed us that there were many reports that had to be filled in during the pandemic, both for the health system and for the COVID-19 task force at the sub-district level.

*"The data was a mess*, *it was a total headache*. *For example*, *some reports are screenshots of images*!*"*

(10BDG2921, Male, Nurse, Urban PHC)

*Culture and communication*. Communication and cultural barriers were reported in rural settings. In Sumba, there are numerous different local languages used in the communities (e.g., Laura, Wewewa, Kodi). Typically, communities had a better understanding when information was delivered in their local languages, rather than Bahasa Indonesia, the national language. Therefore, basic communication was often a challenge for HCWs who were not local, when communicating with the communities and/or providing health education related to the pandemic.

**“***If we have to explain something long enough*, *we ask someone who understands Bahasa Indonesia quite well to translate it into the Kodi language…*. *Yes*, *it’s very different*, *except for the villages that are located near here*, *like Bukambero [a region] to Wewela*, *they have mixed languages*. *Also*, *for the people who live in Wejewa*, *they can understand the Wejewa language only*.*”*

(18SUM913, Female, Health Promotion, Rural PHC)

Another dilemma was regarding the culture of "nose kissing", a form of greeting, and other common greetings, such as hugging. HCWs spoke about the dilemmas they faced between respecting the local culture while also trying to remain a role model and comply with physical distancing for COVID-19 prevention. At times, the HCWs had to decide whether or not to use these greeting forms with their acquaintances. In other instances, the HCWs would tell the community members to withhold the cultural greetings during the pandemic to reduce the likelihood of COVID-19 transmission.

*“Sometimes we told them not to do that [nose kissing]*, *especially among friends and colleagues*. *But it is difficult when we meet our family*, *although we already wear a mask*, *they keep pushing us to nose kiss and hug*.*”*

(13SUM1814, Female, Nurse, Rural PHC)

## Discussion

Based on interview data from a variety of HCWs and related staff at PHCs and hospitals across a range of settings in Indonesia, we established that HCWs were confronted with unprecedented challenges during the first COVID-19 wave. We identified several COVID-19-related role-specific challenges, as well as several cross-cutting challenges. Main findings included challenges in public trust, patient referral issues, lack of training, sub-optimal laboratory capacity, budget and supplies issues, recruiting and retaining staff, and culture and communication. Issues related to trust and mental health were prominent themes across all participant groups from both urban and rural sites and were intertwined with many of the other challenges described. In this study, we highlighted the range of challenges to note the gaps in the system but also to better understand how to support HCWs in the future.

### Importance of trust

In our study, we found trust to be an underlying factor for several challenges faced by HCWs. The public trust (or lack thereof) impacted contact tracing processes, in addition to issues surrounding COVID-19 related stigma. In other contexts, social stigma [9], self and structural stigma perceived by community influenced the contact tracing process, for instance avoiding testing, hiding illness [23], and “marking” people differently [24]. In our findings, some people with COVID-19 infection and their families reacted with resistance when HCWs arrived to their homes in hazmat suits and in an ambulance accompanied by local leader/government to investigate potential COVID-19 cases. Trust further deteriorated when people had asymptomatic COVID-19 and/or did not believe their COVID-19 test results but HCWs were asking them to adhere to social distancing and other public health measures. These findings resonate with studies in the UK and Nigeria that linked lack of trust with government guidelines; although in UK, participants reported high self-adherence to social distancing, they also mentioned how others did not adhere to these measures [25,26], which created confusion and frustration with the public health response [27]. Public trust challenges have been previously noted as a concern in other outbreaks, for example in Ebola where rumors were circulating that the outbreak was not real and was produced for monetary purposes [28]. In our context, we found that the lower levels of public trust reported by HCWs were possibly caused by factors such as frequently changing information, lack of clarity, and varying messages on social media by public figures, experts, government, etc. We also noted low community trust towards the contact tracing policy in Indonesia become the challenge for the HCWs. Our assumption is that the difficulty to gain public trust may not be because of the contact tracing itself but for the fear of subsequent adverse economic and social consequences of the process. Enhancing public trust and providing sustained social capital support for communities could enhance HCWs’ productivity and management of contact tracing and public health measures in general and in future pandemic times. Lessons learned from Ebola included the importance of considering social issues rather than the health issues alone, highlighting that genuine, context-specific public engagement with the community is an essential strategy during a crisis situation to gain public trust. The engagement has to be flexible, adaptable, well-coordinated, and guided by evidence [29].

### Mental health challenges

Another prominent theme across all HCW groups and settings was surrounding mental health. We found that one reason for the mental health challenges, which is in line with recent COVID-19 studies conducted globally [18,30,31], was the constant and rapidly changing information and guidelines. Other studies also found that confusion or misinterpretation and unclear protocols often led to staff stress and weakened self-efficacy [18,32]. As noted elsewhere and within our data, lack of information, unqualified trainings, and increased workload also contributed to confusion regarding changing guidelines [33]. As mentioned above, the gaining of public trust in the community possibly contributed to frustration and stress for HCWs across our sites. Prolonged stress could be a cause of burnout syndrome [34] and our findings support this view. To support the mental health of HCWs, in 2020, the Ministry of Health in Indonesia released detailed mental health guidelines for HCWs [35]. However, in our interviews, we found that some HCWs felt stressed when the support did not meet their expectations, for example access to mental health services or access to basic needs, such as staff incentives and leave time. In order to provide continued support for HCWs, mental health support is mentioned in national guidelines [35] but should be provided at more accessible levels to all HCWs across health center levels, particularly at PHCs, as they are responsible for individual and community-level health.

### Communication and culture

Finally, cultural and communication aspects in rural settings was an important barrier to delivering health education and pandemic-related information. For example, when HCWs provided education to avoid the nose kissing culture [36], it took time for communities to acknowledge that this practice could result in COVID-19 exposure. In fact, the COVID-19 cases in rural settings were relatively low [37], therefore our findings resonate with another study in West Sumba that stated that there were minimal changes in funeral rituals, including the large crowds who gather for the ritual, during COVID-19 [38]. We know from other studies, and our own findings, that different types of healthcare providers and local leaders (e.g. traditional healers, spiritual leaders) are trusted to greater or lesser extents [39]. In order to promote positive education and prevention messages, coordination by trusted individuals and context specific communication would provide better and more trusted information to communities.

### Limitations

Due to mobility restrictions, many interviews were conducted online resulting in connection issues and shorter interview durations for some of the participants. Some interviewees requested that interviews were conducted within the workplace, which may have made them reluctant to speak openly. Additionally, the interviews were only conducted in a limited number of provinces and health care facilities; although the study has made an effort to ensure include diversity in professional roles, health facilities and geographical and socio-economic settings, we cannot rule out the possibility that HCWs in other settings may have had different lived experience and challenges.

## Conclusion and recommendations

Findings of this study outline the various challenges faced by HCWs in the health care system that occurred during the first wave of the COVID-19 pandemic. Supporting HCWs with additional training in soft skills could help with problem solving during complex situations but these trainings should be implemented routinely (i.e. in advance of uncertain situations and on a more regular basis). Based on the cross-cutting challenges across the HCW roles, policymakers should continue the mental health support that is available in national guidelines and make it more accessible for HCWs, particularly at PHCs level. However, we feel that providing mental health services alone is not be enough. Integration of additional organizational support (e.g. mandatory leave time, effective communication of changing information and revised protocols) would also provide security during crisis and in the face of uncertainty. Lastly, a better understanding of local cultures and integration of multiple languages into public health communications would promote inclusion of all communities. Further studies on exploring behaviors on willingness/unwillingness of societies to trust with public health measures, are likely beneficial to deliver better public health messages and prepare for future outbreak preparedness [40].

## Supporting information

S1 File HCW In-depth Interview guide SPEAR Phase 1 Indonesia. (PDF)

S2 File HCW In-depth Interview guide SPEAR Phase 1 English. (PDF)
