# Supplementary material for: Indonesian healthcare professionals’ experiences in rural and urban settings during the first wave of COVID-19: A qualitative study
Source: PLoS One. 2023 Jul 11;18(7):e0288256. doi: 10.1371/journal.pone.0288256 (PMC10335679; doi:10.1371/journal.pone.0288256)
Supplement: S1 File — (PDF) [file pone.0288256.s001.pdf]

Wawancara secara mendalam – wawancara 1 untuk tenaga kesehatan, staf yang berkaitan dengan kesehatan, dan tenaga kesehatan komunitas

Wawancara tatap muka akan berlangsung antara 1-2 jam; wawancara secara daring (*online*) dapat dilakukan melalui serangkaian interaksi daring yang lebih pendek (misalnya wawancara 1 = bagian 1&2, wawancara 2 = bagian 3&4). Pertanyaan dapat diungkapkan ulang bila diperlukan, dan topik serta penelusuran tambahan akan disertakan, berdasarkan pada tanggapan responden. Secara umum, semua topik dalam panduan wawancara ini harus tercakup dalam wawancara tetapi pertanyaan-pertanyaan pastinya tergantung pada keadaan wawancara dan tanggapan dari orang yang diwawancarai.

**Pencair suasana *(ice breaker)***
**Penjelasan dan persetujuan (*Informed consent*)**<br>Pada wawancara pertama, lakukan proses persetujuan lengkap; pada wawancara berikutnya, ingatkan peserta mengenai maksud penelitian dan proses persetujuan. Pada semua kasus, tanyakan peserta apakah mereka memiliki pertanyaan tentang penelitian atau partisipasi mereka dalam studi ini.
**Informasi peserta**
Identitas Peserta<br>Usia<br><br>Pekerjaan<br>Jenis kualifikasi<br>Jumlah tahun sejak kualifikasi<br><br>Tenaga kesehatan masyarakat? Ya/ Tidak
**Wawancara bagian 1: Narasi terbuka tentang pengalaman COVID-19**
Tujuan: mendapatkan cerita pengalaman dari sudut pandang peserta sebelum pertanyaan terfokus lain untuk mendapatkan gambaran cakupan pengalaman mereka.<br><br>1. Untuk memulai, kami ingin mengetahui lebih banyak tentang pengalaman Anda selama wabah COVID-19. Setelahnya, kami akan menanyakan pertanyaan-pertanyaan yang lebih rinci, tapi untuk memulai, mohon ceritakan kepada kami tentang kehidupan Anda selama COVID-19. Silakan mulai cerita Anda dari mana saja dan gunakan waktu sebanyak yang Anda perlukan.<br>2. [setelah mereka selesai] Terima kasih telah berbagi cerita Anda. Saya ingin bertanya lebih kepada Anda tentang [masukkan 1-2 pertanyaan yang ingin Anda telusuri dari cerita mereka].<br><br>[*penelusuran: selama cerita, cobalah untuk tidak menginterupsi untuk perinciannya – catat pertanyaan apa pun yang Anda ingin telusur, gunakan kata-kata yang bersifat pengakuan, seperti ok, ya, mmm dan kalimat penelusuran yang halus seperti “adakah lagi yang ingin Anda tambahkan” atau “apa lagi yang terjadi?” dan kemudian tanyakan pertanyaan tindak lanjut setelahnya.*]
**Wawancara bagian 2: Riwayat pekerjaan, peran dan perubahan tanggung jawab yang dirasakan**

[Topik: riwayat pekerjaan, motivasi untuk mulai bekerja sebagai tenaga kesehatan, kehidupan kerja sehari-hari sekarang dibandingkan dahulu, perubahan besar dalam peran pekerjaan/tugas, persepsi peran dan kontribusi terhadap pandemi

Pertanyaan harus disesuaikan tergantung pada peran mereka (misalnya, tenaga kesehatan/pekerjaan yang berkaitan atau tenaga kesehatan komunitas).]

3. Dapatkah Anda menceritakan tentang karier/ riwayat pekerjaan Anda?
    a. [*jika tidak dijawab]* Berapa lama Anda bekerja sebagai [*cantumkan peran*] di tempat ini?
    b. [*jika tidak dijawab]* Saat ini Anda bekerja di jenis tempat seperti apa?
    c. [*jika tidak dijawab]* Apakah Anda pernah bekerja di tempat lain dengan peran yang sama seperti ini? Jika ya, mohon jelaskan.
    d. [*jika tidak dijawab]* Apakah Anda pernah bekerja sebagai peran pekerjaan lain di tempat ini? Jika ya, mohon jelaskan.

4. Mengapa Anda memutuskan untuk menjadi seorang [*cantumkan peran saat ini atau **TKK***]?

5. Apa yang memotivasi Anda untuk bekerja sebagai [*cantumkan peran*] pada/di [*di spesialisasi/tempat spesifik, misalnya rumah sakit nasional, pusat kesehatan masyarakat, dll.*]?

6. Apa sajakah biasanya tanggung jawab Anda sebagai seorang [*cantumkan peran*] sebelum COVID-19?

Sekarang saya memiliki sedikit pertanyaan tentang seperti apa pekerjaan Anda sekarang ini. *[Jika saat ini tidak terdapat transmisi komunitas aktif, tanyakan tentang rincian saat wabah aktif. Untuk TKK, tanyakan tentang pengalaman terkait pekerjaan TKK.]*

7. Seperti apakah biasanya hari kerja Anda sekarang? Apakah berbeda dengan sebelum COVID-19? Jika ya, seperti apa? [*alternatif: bagaimanakah COVID-19 telah mengubah aktivitas kerja Anda?]*

8. Apakah telah terjadi perubahan besar dalam peran atau tugas pekerjaan Anda sejak/selama COVID-19?

    a. [*Jika tidak dijawab*] Apakah Anda mendapatkan tugas tambahan yang dibebankan pada beban kerja normal Anda? Jika ya, apakah tugas tambahan tersebut dan kenapa? [*penelusuran: karena tugas baru atau tugas yang diambil alih dari orang-orang lain yang lebih terlibat dalam respons COVID-19 , dll.]*
    b. Apakah terdapat tugas yang tidak lagi diprioritaskan secara umum?
        i. Jika ya, tugas apakah?
        ii. Jika ya, mengapa menurut Anda tugas tersebut tidak diprioritaskan?

c. Apakah ada tugas yang dialokasikan kepada orang lain atau departemen lain? Jika ya, tugas apakah dan kepada siapa?

9. Apakah wabah COVID-19 telah berdampak terhadap pekerjaan Anda dengan cara lain, di luar perubahan pada peran pekerjaan? Jika ya, bagaimana? [*penelusuran: tuntutan pekerjaan vs rumah, stres tambahan, dll. –dapat berhubungan dengan diskusi pada q1]*

10. Apakah tantangan terbesar Anda saat ini?

11. Strategi apakah, jika ada, yang sudah Anda (dan rekan atau pimpinan Anda) ciptakan untuk memitigasi tantangan tersebut?

12. Apa yang Anda rasakan tentang masuk ke dalam komunitas Anda atau tempat pekerjaan Anda selama COVID-19?
    a. [*jika tidak dijawab*] Apakah risiko utama dalam melakukan pekerjaan Anda?
    b. [*jika pandemi sudah teratasi*] Apa yang Anda rasakan sekarang masuk ke dalam komunitas Anda?

13. Apakah Anda memiliki akses terhadap pasokan APD yang memadai untuk melakukan pekerjaan Anda?
    a. Jika ya, apakah seperti ini sepanjang wabah COVID-19?
    b. Jika tidak, bagaimanakah hal ini berdampak terhadap pekerjaan Anda?
    c. [cantumkan pertanyaan spesifik sesuai negara dan pertanyaan terkait temuan survei]

14. Seberapa siapkah Anda dalam hal pelatihan atau bimbingan COVID-19 (untuk tenaga kesehatan komunitas, tentang penyampaian pesan kesehatan)?

15. Bagaimanakah Anda melihat kontribusi Anda sebagai seorang [*cantumkan peran*] selama wabah?

Wawancara bagian 3: Dampak pekerjaan terhadap keluarga dan komunitas

Sekarang kami memiliki sedikit pertanyaan tentang dampak COVID-19/peran pekerjaan terhadap keluarga Anda dan komunitas.

16. Bagaimanakah situasi terkini perumahan Anda?
    a. Apakah hal ini berubah sejak sebelum/ selama COVID-19? Jika ya, bagaimana?
    b. [*jika ya*] Apakah perubahan tersebut memiliki dampak (positif atau negatif atau keduanya) terhadap Anda dan keluarga Anda?
        i. Jika ya, dapatkah Anda jelaskan?

17. Apakah menurut Anda tenaga kesehatan (termasuk mereka yang tidak bekerja langsung menghadapi pasien COVID-19) sebaiknya sementara tinggal di tempat terpisah dari keluarga mereka? Mengapa atau mengapa tidak? [*selidikilah untuk contoh-contoh, jika memungkinkan*]

18. Apa yang keluarga Anda rasakan tentang Anda yang bekerja di layanan kesehatan/ atau berhubungan dengan kesehatan pada saat ini?
    a. Apakah hal tersebut sama atau berbeda dari sebelum/ selama COVID-19? Jika berbeda , bagaimana?

19. Bagaimana menurut Anda COVID-19 berdampak terhadap perilaku mencari layanan dari orang-orang dalam komunitas Anda? [*selidikilah jika hal ini telah berubah selama fase-fase berbeda dari pandemi untuk negara tertentu dan selidikilah untuk contoh-contoh spesifik*].
20. Apakah ada dampak lain yang dirasakan Keluarga Anda karena tugas Anda saat ini sebagai tenaga Kesehatan atau staff terkait?
21. Bagaimana menurut Anda COVID-19 berdampak secara luas pada komunitas/masyarakat (contoh: apa yang membuat seseorang menjadi sangat rentan atau kurang rentan (atau sangat terdampaka atau sedikit terdampak?)

Wawancara bagian 4: Stigma sosial dan pengucilan/'*othering*' yang berhubungan dengan COVID-19, respons tingkat nasional

22. Apakah ada kelompok spesifik yang lebih bertanggung jawab terhadap penyebaran COVID-19 di [*cantumkan lokasi negara*] (atau di tempat lain, jika tak ada penyebaran)?
    a. Jika ya, siapakah kelompok ini?
    b. Mengapa Anda berpikir mereka lebih bertanggung jawab terhadap penyebaran COVID-19?

23. Dibandingkan negara-negara lain, menurut Anda apakah strategi terbaik yang telah [*cantumkan lokasi negara*] masukkan dalam respons mereka terhadap COVID-19? Jelaskan.

24. [*cantumkan pertanyaan-pertanyaan tambahan spesifik sesuai dengan negara bilamana sesuai: pertanyaan terkait etik untuk mereka yang terlibat dalam uji klinis, atau respons terhadap pertanyaan spesifik]*

Penutup

25. Apakah ada topik lainnya yang ingin Anda diskusikan dengan saya hari ini?
