# Supplementary material for: Indonesian healthcare professionals’ experiences in rural and urban settings during the first wave of COVID-19: A qualitative study
Source: PLoS One. 2023 Jul 11;18(7):e0288256. doi: 10.1371/journal.pone.0288256 (PMC10335679; doi:10.1371/journal.pone.0288256)
Supplement: S2 File — (PDF) [file pone.0288256.s002.pdf]

In-depth interview – health care workers, health related staff, and community health workers interview 1

In-person interviews will last between 1-2 hours, online interviews may be conducted over a series of shorter online interactions (e.g. interview 1 = parts 1&2, interview 2 = parts 3&4). Questions might be re-phrased, when necessary, and additional topics and probes will be included, based on the responses of the interviewees. In general, all topics in this interview guide should be covered in the interview(s) but the exact questions will depend on the nature of the interview and responses of the interviewee.

Ice breaker
Informed consent<br>If first interview, conduct full consent process; if subsequent interview, remind participant about nature of study and consent process. In all cases, ask participant if they have any questions about the study or their participation in this study.
Participants' information
Participant ID<br>Age<br><br>Occupation<br>Type of qualification<br>Number of years since qualification<br><br>Community health worker? Yes / No<br><br>Have you been tested for COVID-19?<br>If yes, what was the result?
Interview part 1: Open ended narrative of COVID-19 experiences
Goal: to gain a narrative of experiences from the participant's point of view prior to any focused questions to gain a picture of what their experiences include<br><br>1. To start, we would like to know more about your experiences during the COVID-19 outbreak. After, we will ask more focused questions but to begin, please tell us the story of your life during COVID-19. Please start the story where you like and take as much time as you need.<br>2. [*when they are finished*] Thank you for sharing your story. I would like to ask you more about [*insert any questions that you would like to probe on from their story*].<br><br>*[probe: during the narrative, try not to interrupt for details – note any questions you would like to probe on, use acknowledgement probes, e.g. ok, yes, mhhh and gentle probes, e.g. "is there anything else you would like to add" or "what else happened?" and then ask follow-up questions after.]*
Interview part 2: Career history, role and perceived changes in responsibility

[Topics: career history, motivations to start working as a health worker, daily work life now versus in past, major changes in job role/duties, perception of role and contribution to pandemic

Questions should be adapted depending on their role (e.g. health care worker/related work or community health worker).]

3. Could you tell me about your career/job history?
    a. [*if not answered]* How long have you been working as a [*insert role*] in this setting?
    b. [*if not answered]* What type of setting do you currently work in?
    c. [*if not answered] H*ave you worked in other settings in this same role? If yes, please explain.
    d. [*if not answered]* Have you worked in other job roles in this setting? If yes, please explain.

4. Why did you decide to become a [*insert current role or **CHW***]?

5. What motivated you to work as [*insert role*] a/in [*in specific specialty/setting, e.g. national hospital, community health center, etc*]?

6. What were your usual responsibilities as a [*insert role*] prior to COVID-19?

7. In general, what have been the most challenging moments in your career [*for CHW]* or within your time as CHW?

8. How did you overcome these challenges?

Now I have a few questions about what your job is like now.
[If no active current community transmission, ask about specifics during the active outbreak. For CHW, ask about experiences related to CHW work.]

9. What is a typical day like at your work now? Is that different from before COVID-19? If yes, how? [*alternative: how has COVID-19 changed your work activities?]*

10. Have there been major changes in your <u>job role or duties</u> since/during COVID-19?

    a. [*If not answered*] Have you had additional duties added on to your normal workload? If yes, what were they and why? [*probe: because of new tasks or tasks taken over from others who are more involved in COVID-19 response, etc]*
    b. Are there duties/tasks that are no longer prioritized in general?
        i. If yes, what are the duties?
        ii. If yes, why do you think they are not prioritized?
    c. Have duties been allocated to other people or departments? If yes, what duties and to whom?

11. Has the COVID-19 outbreak impacted your work in other ways, beyond changes in job role? If yes, how? [*probe: demands of work vs home, added stress, etc – can link to discussions in q1]*

12. What are the top challenges for you at this moment?

13. What strategies, if any, have you (and your colleagues or leaders) created to mitigate these challenges?

14. How do/did you feel going into your community or into your workplace during COVID-19?
    a. [*if not answered*] What are/were the major risks in performing your work?
    b. [*if pandemic is contained*] How do you feel now going into your community?

15. Do you have access to sufficient supplies of PPE to conduct your job?
    a. If yes, has this been the case throughout the COVID-19 outbreak?
    b. If no, how did this impact your job?
    c. [insert country specific questions and questions related to survey findings]

16. How prepared do you feel in terms of training or guidance related to COVID-19 (for community health workers, about health messaging)?

17. How do/did you see your contribution as a [*insert role*] during the outbreak?

Interview part 3: Impact of job on family and community

Now we have a few questions about the impact of COVID-19/job role on your family and the community.

18. What is your current housing situation?
    a. Has this changed since before/during COVID-19? If yes, how?
    b. [*if yes*] Has the change had any impact (positive or negative or both) on you and your family?
        i. If yes, could you explain?

19. Do you think health care workers (including those who do not work directly with COVID-19 patients) should temporarily live in a space away from their families? Why or why not? [*probe for examples, if possible*]

20. How does your family feel about you working in a healthcare/or health related role at this time?
    a. Is that the same or different from before/during COVID-19? If different, how?

21. How do you think COVID-19 is impacting [*or impacted*} people in your community care seeking behaviors? [*probe if this has changed during the different phases of the pandemic for the specific country and probe for specific examples*].

Interview part 4: Social stigma and 'othering' associated with COVID-19, country-wide responses
22. Are there specific groups who are more responsible for the spread of COVID-19 in [*insert country where located*] (or elsewhere, if no spread)?<br>a. If yes, who are these groups?<br>b. Why do you think they are more responsible for the spread of COVID-19?<br><br>23. What do you think are the best strategies to prevent COVID-19?<br>a. What are the biggest challenges the community has in achieving these prevention measures?<br>b. Are there communities that have more or less challenge [*for with each strategy they list*]?<br><br>24. Compared to other countries, what do you think are the best strategies that [*list country where located*] has included in their response to COVID-19? Explain.<br><br>25. [*add additional country specific questions as appropriate: ethics related questions to those involved in clinical trials, or response specific questions]*
**Closing**
26. Are there any other topics you would like to discuss with me today?
